# The Biochemical and Genetic Basis for the Biosynthesis of Bioactive Compounds in *Hypericum perforatum* L., One of the Largest Medicinal Crops in Europe

**DOI:** 10.3390/genes11101210

**Published:** 2020-10-16

**Authors:** Paride Rizzo, Lothar Altschmied, Beena M. Ravindran, Twan Rutten, John C. D’Auria

**Affiliations:** Leibniz Institute of Plant Genetics and Crop Plant Research (IPK), 06466 Gatersleben, Germany; rizzo@ipk-gatersleben.de (P.R.); lothar@ipk-gatersleben.de (L.A.); ravindran@ipk-gatersleben.de (B.M.R.); rutten@ipk-gatersleben.de (T.R.)

**Keywords:** *Hypericum perforatum*, Saint John’s Wort, hypericin, hyperforin, dark glands, pale glands, biosynthesis

## Abstract

*Hypericum perforatum* L. commonly known as Saint John’s Wort (SJW), is an important medicinal plant that has been used for more than 2000 years. Although *H. perforatum* produces several bioactive compounds, its importance is mainly linked to two molecules highly relevant for the pharmaceutical industry: the prenylated phloroglucinol hyperforin and the naphtodianthrone hypericin. The first functions as a natural antidepressant while the second is regarded as a powerful anticancer drug and as a useful compound for the treatment of Alzheimer’s disease. While the antidepressant activity of SJW extracts motivate a multi-billion dollar industry around the world, the scientific interest centers around the biosynthetic pathways of hyperforin and hypericin and their medical applications. Here, we focus on what is known about these processes and evaluate the possibilities of combining state of the art omics, genome editing, and synthetic biology to unlock applications that would be of great value for the pharmaceutical and medical industries.

## 1. Introduction

**Figure 1:**
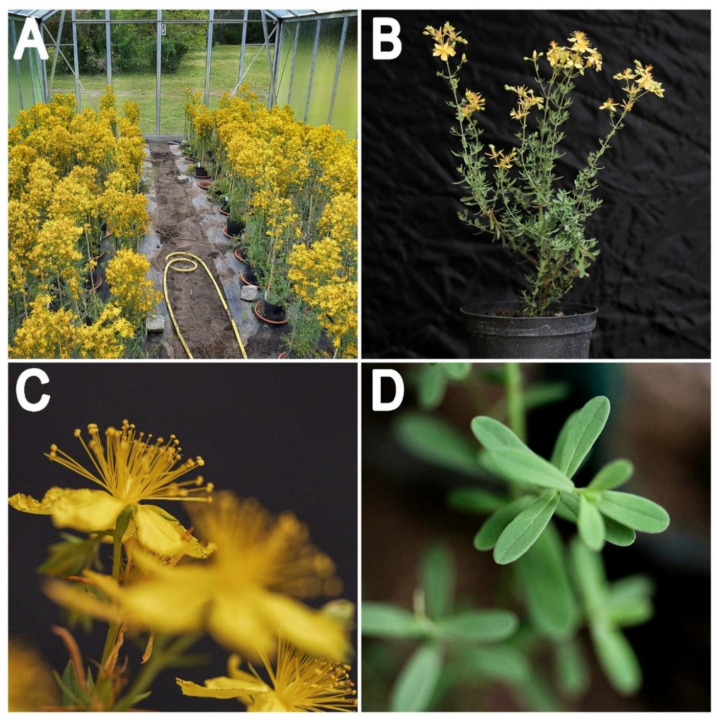
Canopy and appearance of plants of Hypericum perforatum. (**A**): Greenhouse cultivation of a sexual diploid genotype (HyPR-01); (**B**): Single plant 7 months old in full flowering; (**C**): Detail of open flowers; (**D**): Detail of adult leaves. Photos by Paride Rizzo.

*Hypericum perforatum* (Figure 1) belongs to the order Malpighales, which includes more than 16,000 species. Representatives of the genus *Hypericum* (469 species) can be found in every temperate climate around the globe and comprise species with a wide variety of plant architecture ranging from small herbs to trees [1].

Extracts of Saint John’s Wort SJW have been used for centuries to treat anxiety, depression, sciatica, or even wounds [2,3,4]. In recent years, SJW extracts have been used mainly for their antidepressant properties. Numerous behavioral studies on humans and rats have been performed and have shown their efficacy [5,6,7,8]. This has resulted in a multi-billion dollar market for *Hypericum*-based products that consists of more than 13% of the total herbal supplement trade in Europe [1] and approximately 6 billion USD worldwide [9]. While the antidepressant properties of *Hypericum* extracts are linked to the presence of hyperforin, another major component, the naphtodianthrone hypericin has been identified as a promising anticancer agent and a potential treatment against neurodegenerative diseases including Alzheimers’s disease [10,11]. To harness the full medical potential of these compounds, identification of genes involved in their biosynthesis is required, since novel genome editing tools and approaches in synthetic biology provide routes to production in genetically modified microorganisms and plants. Several genes potentially involved in the biochemical pathways to hyperforin and hypericin have been characterized, but functional proofs are missing in many cases and neither of the two pathways is fully understood [12,13,14]. In this review, we describe the most current state of knowledge regarding the genes and enzymes that participate in the biosynthesis of these important pharmaceuticals as well as their medicinal uses.

## 2. The Relevance of Hyperforin, Hypericin and Other Bioactive Compounds from *Hypericum perforatum* L.

The composition of extracts from *H. perforatum* has been studied extensively and many secondary metabolites including rutin, hyperforin, hyperoside, quercitrin, isoquercitrin, quercetin, hypericin, and chlorogenic acid, have been identified [3,15,16,17]. Nevertheless, two compounds have emerged as the most important for the pharmaceutical industry: hyperforin and hypericin.

Hyperforin is a prenylated phloroglucinol derivative that constitutes the most abundant lipophilic component of the hydroalcoholic extracts of *H. perforatum* [6,18,19]. The antidepressant and neuro-active effects for which SJW extracts are known could eventually be attributed to the presence of this particular component and pure hyperforin alone can reproduce most of them [6,18,19]. Many antidepressants rely on the capacity to inhibit the uptake of important neurotransmitters like serotonin, dopamine (DA), or noradrenaline (NA) [20]. Hyperforin constitutes a natural synaptosomal inhibitor of neurotransmitter uptake. Its efficiency is comparable to that of tricyclic antidepressants (TCA) or serotonin specific inhibitors (SSRI) but without the side-effects typical for these drugs [6].

Unlike many other anti-depressants, hyperforin can inhibit the synaptosomal uptake of the amino acid transmitters, gamma-aminobutyric acid (GABA), and L-glutamate [21]. This is due to its mode of action that instead of being based on the competitive interactions for the transporter binding sites, relies on the increase of intracellular Na^+^ known to be critical for the regulation of neurotransmitter uptake [21,22,23]. These characteristics confer to hyperforin the status of a broad-spectrum uptake inhibitor of neurotransmitters, whose effects have been reported in several behavioral studies on rats and humans [5,6,7,8].

While SJW extracts are commonly sold as mild antidepressants, reports on their efficiency are contradictory, with some studies finding no enhanced effect over placebo treatments [24,25]. In addition, there are reports regarding the existence of local bias being observed in German-speaking countries [26]. Besides its use as a potential antidepressant, hyperforin is also considered a powerful antibacterial compound, effective against all gram-positive bacteria and penicillin-resistant (PRSA) and methicillin-resistant (MRSA) *Staphylococcus aureus* [27].

SJW extracts are also the main source of natural hypericin. This naphtodianthrone is regarded as an efficient anticancer compound that induces an apoptotic response after specifically binding to melanoma cancer cells [28]. The successful application of hypericin in cancer photodynamic therapy (PDT) takes advantage of the properties of hypericin as one of the most powerful photosensitizers known in nature [11]. When exposed to light, hypericin can induce an apoptotic signal. This involves the formation of reactive oxygen species (ROS) that eventually lead to the killing of the tumor [29,30]. Hypericin therapy also induces an increase in cytokine levels, leading to an inflammatory response and the activation of immune cells [11,31]. This mechanism is known as Immunogenic Cell Death (ICD). Hypericin is classified as a type II immunogenic cell death inducer [32] since the apoptotic response it induces is triggered at the level of the endoplasmic reticulum. Molecules capable of inducing such immunogenic cell death responses are classified as “damage-associated molecular patterns” (DAMPs) and have become a new avenue of treatment for cancer [32]. Hypericin is the most potent natural ICD inducer known to date, and its properties are being explored to find even more effective analogues.

The antiviral properties of hypericin were shown against a large number of viruses including HIV, influenza virus A, herpes simplex, bovine diarrhea virus (BVDV), hepatitis C, duck reovirus, and bronchitis virus [33,34,35,36,37,38,39,40]. Hypericin seems to be particularly effective against enveloped viruses by targeting and modifying viral proteins [34]. This effect is enhanced by light, which not only inactivates the virus, but can also prevent fusion of the virus with the cell membrane of the host [41]. 

Interest in SJW extracts has gone beyond the antidepressant, anti-cancer and antiviral applications after it was discovered that these extracts can reduce both memory impairment and β-amyloid fibril deposition in the brain of APP-transgenic mice [10]. These results are compliant with in vitro studies showing that hypericin can interfere and significantly inhibit the formation of β-amyloid fibrils by associating with the precursors of these fibrils [42,43]. At high concentrations, hypericin can alter cell membrane permeability and induce anomalous functioning in the affected cells [42,44,45]. Rats treated with SJW extracts also showed an increased expression of the ABCC1 transporter which is involved in the clearance of brain tissues from the β-amyloid plaques [10]. Since this effect is independent of hyperforin it suggests an additional role for hypericin [10].

In light of these recent studies, it is likely that there will be an increased pharmaceutical demand for both hyperforin and hypericin. Since large-scale synthetic production is prohibitively expensive, SJW extracts remain the main source of these two compounds. Unraveling their biosynthetic pathways would not only allow the creation of multi-tasking SJW plants producing customized levels of bioactive compounds, but also be used for engineering microorganisms producing these, and related, valuable drugs on a large scale.

It is important to note that other compounds produced by *H. perforatum* are also regarded as bioactive compounds. These include phenolic derivatives such as tannins and xanthones. Further examples include the flavonoids quercetin, hyperoside, rutin and isoquercitrin. Concentrations of flavonoids can range between 7 to 12% in flowers [3,9]. Flavonoids confer strong antioxidative properties to SJW extracts. When in high concentrations, these extracts can be referred to as FEHP (Flavonoids-rich extracts of *H. perforatum*). They are proposed to reduce the effects of oxidative stress that acts upon such processes as aging, carcinogenesis, diabetes, and many other clinical conditions [46]. Flavonoids like quercitin, kaemferol, and biapigenin also have gastro and neuroprotective functions due to their capacity of inhibiting the peroxidation of mitochondrial membranes. By maintaining mitochondrial transmembrane electric potential, the overload of calcium uptake in the mitochondrion is avoided [47].

## 3. Secretory Structures of *Hypericum perforatum*

**Figure 2:**
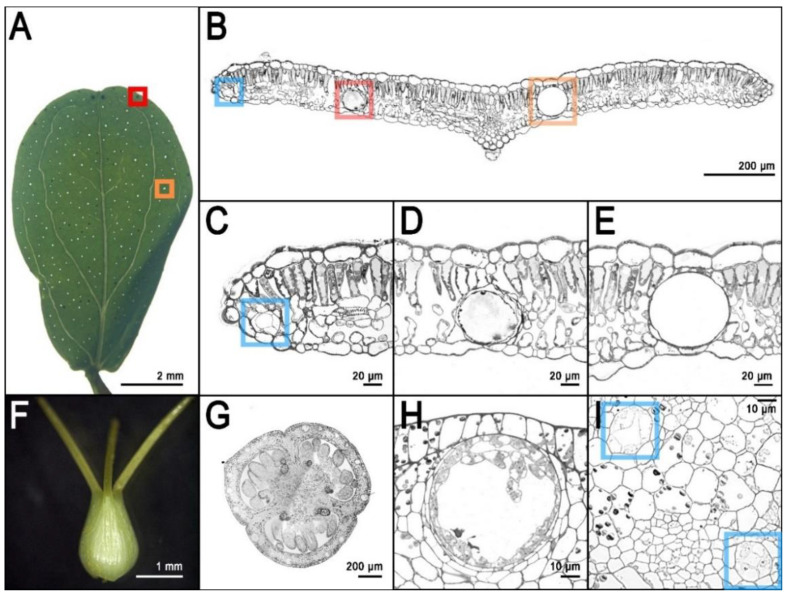
Secretory structures of Hypericum perforatum. (**A**): Adult leaf in backlight showing dark glands (red square) and pale glands (orange square); (**B**): Cross section of an adult leaf showing one pale gland (orange square), one dark gland (red square) and a type c secretory canal (blue square); (**C**): Detail of the type C canal from photo B (**D**): Detail of dark gland from photo B; (**E**): Detail of pale gland from photo B; (**F**): Pistil from open flower (external view); (**G**): Cross section of a pistil from an open flower showing dark glands growing on the placental tissue and type C secretory canals in the external portion of every carpel; (**H**): Detail of a type C canal from photo G; (**I**): Detail of type A canals from the placental tissue in photo G. Photos by Twan Rutten and Paride Rizzo.

*H. perforatum* (and many other species of the genus *Hypericum*) is characterized by different types of secretory structures that are specialized in the storage of specialized metabolites and are distributed in all reproductive and vegetative tissues of the plant. The pale glands, also known as translucent glands, are especially concentrated in the leaf lamina (Figure 2A,B,E). The presence of these glands causes the leaves to appear to be perforated when observed in backlight (Figure 2A), hence the name *perforatum*.

Pale glands are known to be the specialized organs for the storage of high quantities of hyperforin [48]. Light-microscopy observations of cross sections of leaves stained with toluidine blue reveal many pale glands embedded in the parenchyma and delimited by two layers of cells. Of these layers, the external one appears thicker due to modified parenchyma cells, while the layer that surrounds the lumen is made by flattened cells with very thin cell walls [49].

*H. perforatum* is also characterized by other translucent secretory structures called canals because of their oblong shape. There are three types of canals: A (Figure 2B,C,I), B, and C (Figure 2H). Type A canals occur in every part of the plant but the stamens and they are characterized by a lumen that is much smaller than the one from the pale glands and is delimited by 4 polygonal cells (Figure 2I). Type B canals are present in several floral organs and the stem. Their structure is similar to the pale glands, in which their main difference is their oblong shape. Finally, type C (Figure 2G,H) canals have only been described in the carpels of *H. perforatum.* Type C canals show one or multiple layers of intensely stained secretory cells with thin walls that surround a large lumen [14,49].

**Figure 3:**
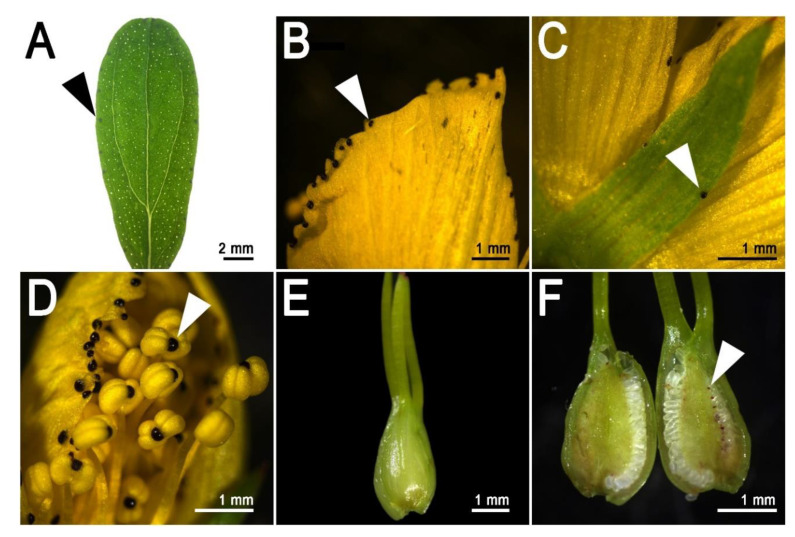
Dark glands in different epigeal parts of Hypericum perforatum. (**A**): Adult leaf; (**B**): Dark glands on the petal rim; (**C**): Dark glands on the sepal rim; (**D**): Dark glands in the anthers; (**E**): Pistil external view, no dark glands visible; (**F**): Dark glands on the surface of the placental tissue. Short arrows indicate the dark glands. Photos by Paride Rizzo.

The secretory structures described above, especially the pale glands, are the storage organs for hyperforin, adhyperforins, other phloroglucinols as well as a variety of alkaloids, lipids, resins, and essential oils [17,49]. Nevertheless, the naphtodianthrones such as hypericin are stored in other dedicated secretory/storage organs called dark glands (Figure 2B,D) [50]. These structures are easily detectable in several epigeal parts of *H. perforatum* and other species of the genus *Hypericum* (Figure 3).

**Figure 4:**
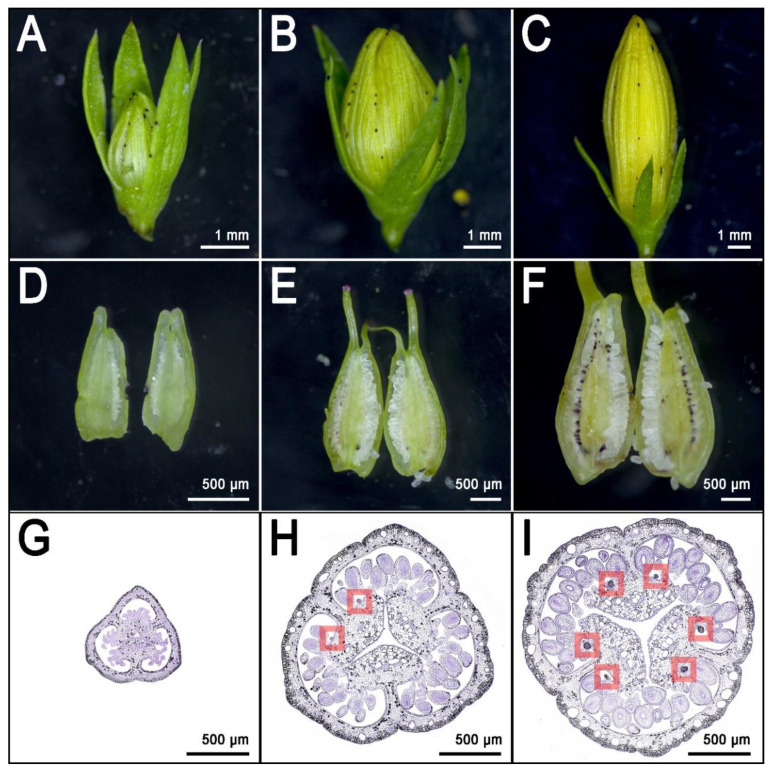
Multiple stages of flower development (upper row) with corresponding pistils shown as longitudinal sections (middle row) and thin transverse sections (lower row). (**A**): Flower bud of length 2.14 mm; (**B**): Flower bud of length 5.24 mm; (**C**): Flower bud of length 10.38 mm; (**D**): Longitudinally sectioned pistil from flower bud in photo A; (**E**): Longitudinally sectioned pistil from flower bud in photo B; (**F**): Longitudinally sectioned pistil from flower bud in photo C; (**G**): Transverse section of a pistil before the differentiation of dark glands. This stage is completely dark glands free; (**H**): Cross transverse section of a pistil right at the moment of the differentiation of dark glands from the placental tissues. Dark glands highlighted by red squares; (**I**): Transverse section of a pistil with well differentiated and growing placental dark glands. All 6 rows (2 per carpel) of dark glands are visible on the same section and highlighted by red squares. Photos by Paride Rizzo and Twan Rutten.

The morphology of dark glands has been accurately described in the foliar tissues, where they are mainly (but not exclusively) positioned along the leaf rim (Figure 3A) and embedded in the mesophyll with two layers of flattened cells defining the lumen of the gland [51]. The glandular cells show modifications similar to the ones observed in the meristematic cells, which includes large nuclei and nucleoli. In addition, the glandular cells contain an accumulation of vesicles between cells, and also contain dictyosomes and active Golgi apparatus inside the cells of the inner glandular layer [51]. The vesicles seem to carry out the transport mechanisms necessary for secretory processes, and they seem to travel mainly through the symplast. Dark glands were also recently characterized in the reproductive tissues of *H. perforatum* (Figure 3F; Figure 4F,I) where they occur in high numbers (up to ~130), especially in the placental tissue [14]. In these tissues, the dark glands can reach a length at maturity of up to >100 µm. The placental dark glands show clear morphological differences with the foliar ones. They are not embedded in the placental tissue, rather growing on its surface like protuberances (Figure 2G; Figure 4I) that are formed as a result of the differential growth rate of some epidermal cells (schizogenous development). Furthermore, the lumen of these glands is delimited by a single layer of flattened epidermal cells unlike the double layer morphology in the leaves [14].

## 4. The Frontier of Dark Gland and Hypericin Biosynthesis Research

The dark glands of *H. perforatum* caught the attention of generations of scientists who tried to unravel the mechanisms of hypericin biosynthesis. With the advent of omics technologies, it is possible to study for the first time the transcriptome of a dark gland, and from that information, begin to infer the genes involved in hypericin biosynthesis. Historically, the scientific community addressed the leaf as a model tissue for this kind of study. Dark glands in the leaves are mainly (but not exclusively) distributed along the rim. However, it is difficult to find contrasting phenotypes of *H. perforatum* in which a glandless leaf can be compared with a glanded one. Furthermore, the foliar dark glands differentiate already at very early stages of leaf development and this makes it difficult to address stages without glands (pre-differentiation) with post-differentiation stages. These issues resulted in studies which compared the transcriptome of the leaf rim with the leaf lamina [13,52]. Although these two tissues are part of the same organ, they have different anatomies and patterning. This constitutes a bias for any comparison between these two different fractions of the leaf. Nevertheless, RNAseq experiments including the rim vs lamina comparison, represented the first glimpse into the transcriptome of dark glands, and also identified putative candidate genes involved in the biosynthesis of hypericin. In the most recent years, the pistil has emerged as a more suitable model for this kind of study [14]. The pistil of *H. perforatum* is in many cases heavily glanded, with up to >130 glands per pistil and is distributed along six rows on the surface of the placental tissue (Figure 4I).

**Figure 5:**
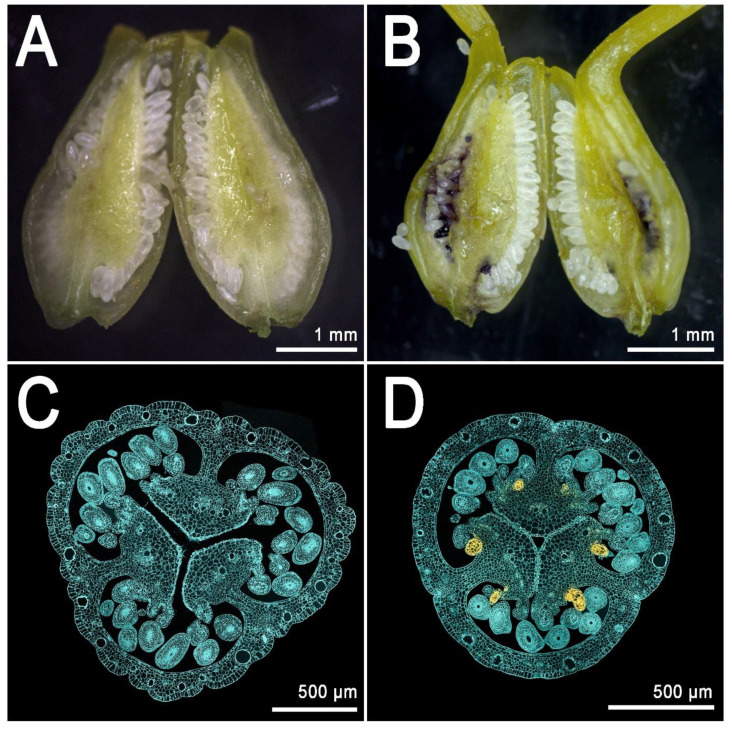
Comparison between contrasting (glanded and glandless) phenotypes of pistils of *Hypericum perforatum*. (**A**): Transverse section of a glandless pistil from an open flower of the genotype H06-1877; (**B**): Transverse section of a glanded pistil from on open flower of the genotype HyPR-09. Dark glands are visible on the surface of the placental tissue and under the layer of (white) developing ovules. (**C**): Cross section of a pistil (genotype H06-1877) observed under the confocal microscope. No hypericin fluorescence is detected. (**D**): Cross section of a pistil (genotype H06-1877) observed under the confocal microscope. Hypericin fluorescence signal (in yellow) is localized in the placental dark glands. Photos by Twan Rutten and Paride Rizzo.

These glands are larger than their foliar counterpart and their density is much higher than in the leaf, which normally shows a maximum of ~20–30 glands [14]. The placental glands do not differentiate until the flower buds reach an average length of ~5 mm [14] (Figure 4E,H; dark gland differentiation stage). This means that during early flower development, the placentas are completely glandless (Figure 4D,G) and can be used as pre-differentiation stages in the context of multi-stage experiments dealing with the development of dark glands. Furthermore, a recent study which characterized the floral development of 93 genotypes, identified a combination of perfectly contrasting phenotypes in which some SJW accessions never revealed any dark glands in their pistils (Figure 5A,C) in contrast to other accessions in which pistils were heavily glanded (Figure 5B,D) [14].

These contrasting morphologies corresponds to equally contrasting metabolic phenotypes. This means that the heavily glanded pistils show high levels of hypericin and related precursors, while the glandless pistils are characterized by the absence (or traces) of these compounds. In light of these results, the pistil represents a nearly ideal platform for the study of dark gland development and hypericin biosynthesis.

The gene expression results reported by Rizzo et al. identified clusters of genes following different temporal dynamics of gene expression [14]. Some genes coding for enzymes putatively involved in the biosynthesis of hypericin are expressed mainly after the differentiation and growth of dark glands. The combination of spatio-temporal expression and annotation on heterologous systems makes these genes valuable for the characterization of this complex biosynthetic pathway (see the following section). Another small cluster of genes was reported to have a peak of expression strictly synchronized with the differentiation of the first dark gland primordia in the placenta (Figure 4H). Only two transcription factors are part of this cluster. *MYB38* is an R2R3-Myb gene that belongs to the subgroup 14 of the Myb family [53]. All the members of this subgroup are exclusively involved in cell differentiation and organ identity [54,55,56,57]. *MYB38* is the orthologue of *RAX2* from *Arabidopsis thaliana*, and is known to be involved in developmental processes by regulating lateral patterning and lateral meristem initiation [55]. This is a process that resembles the lateral patterning in placental tissues of *H. perforatum*, from which dark glands originate. The only other transcription factor with a nearly identical expression pattern is an ortholog of *A. thaliana AGL6* (Agamous Like 6), a MADS-box gene responsible for the determination of organ identity and meristem differentiation in the flower [58]. *AGL6* is also known to be the MADS-box gene with the highest rate of duplication and neofunctionalization in the evolutionary history of dicotyledons [59]. These two genes have very low levels of expression in placental tissues that never differentiate dark glands, while they are 100–1000 times more highly expressed in glanded placental tissues, starting right at the moment of dark gland primordia differentiation [14]. In light of their expression pattern and previously reported annotations, *MYB38* and *AGL6* constitute the most robust candidates for the hyperactivation or shutdown of the dark glands in *H. perforatum.*

The transcriptomic data derived from placental tissues not only identified candidates for hypericin biosynthesis and dark gland formation, but also provides a set of candidate genes encoding for transporters normally seen during vesicle-related trafficking [14]. This contributes to the definition of an integrated model in which proposed biochemical pathways are integrated with developmental dynamics, morphological information, developmental dynamics, metabolomics, and transcriptomics data, as well as functional annotation and protein localization. According to this model, the transport of naphtodianthrones could follow a mechanism similar to flavonoid transport, which relies on vesicles associated with glutathione-S-transferases (GST) [60]. The presence of vesicles associated with dark glands was documented in the past and was further validated by more recent results [14,51].

## 5. Hypericin Biosynthesis

The biochemistry of hypericin originated with Buchner, who named it ‘Hypericumrot’ [61]. After hypericin had been isolated from *Hypericum perforatum* by Černý *et al.*, its structure was demonstrated through chemical synthesis by Brockmann et al. [62,63]. In parallel to the chemical synthesis, Brockmann’s group developed a hypothesis for the biochemical pathway in plants [64,65]. The well-understood acetate malonate pathway, which occurs in many plant, fungal, and animal organisms was assumed to lead to an octaketide (3) through condensation of one acetyl-CoA (1) and seven malonyl-CoAs (2). Multiple aldol cyclizations, well known from chemical synthesis, would convert the octaketide into emodin anthrone (8). The oxidative dimerization of emodin anthrone would then lead, via emodin dianthrone and protohypericin (11) to the final product hypericin (12) [66].

**Figure 6:**
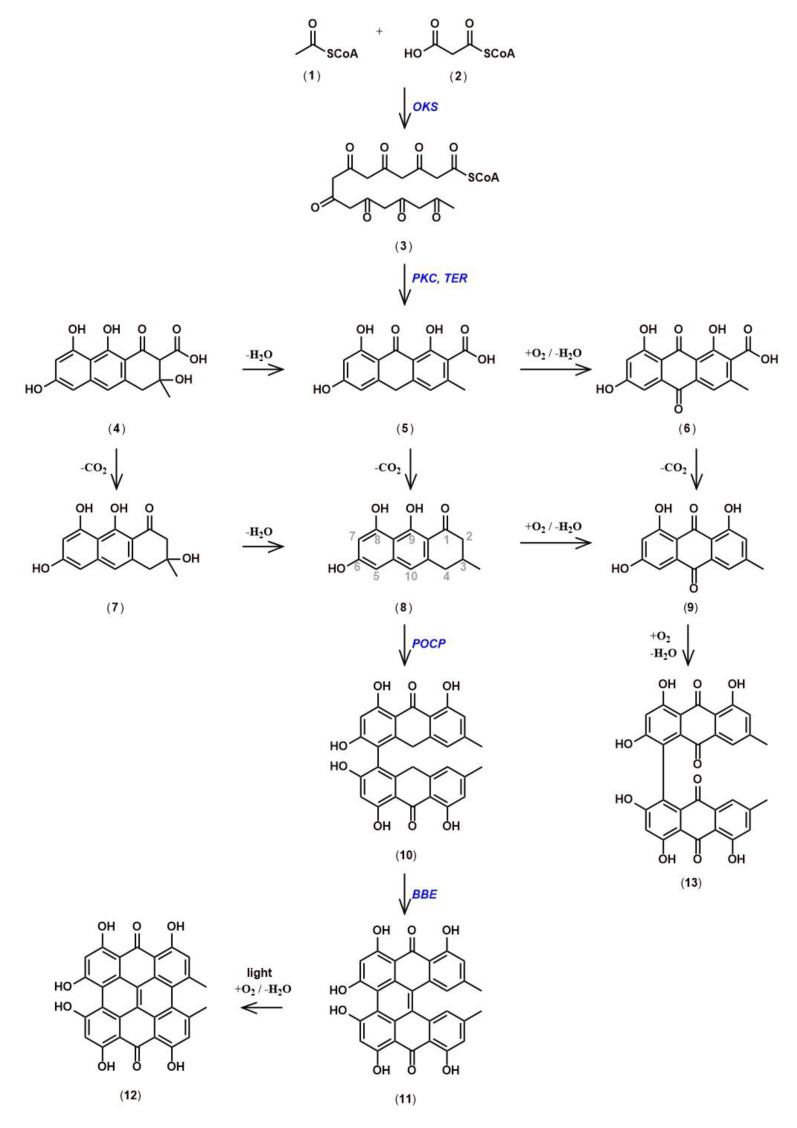
The hypothetical pathway for the biosynthesis of hypericin: Enzymes, represented in blue are suggested by previous literature.: *OKS*, octaketide synthase; *PKC*, polyketide cyclase; *TER*, thioesterase; *POCP*, phenoloxidative coupling protein; BBE, berberine bridge enzyme.

Based on this hypothetical pathway (Figure 6), Bais et al. reported the isolation of a gene called *Hyp-1* from *H. perforatum* with sequence homology to pathogenesis related-10 (*PR-10*) genes [67,68]. Hyp-1 protein was reported to convert emodin (9), an oxidation product of emodin anthrone, into hypericin in the dark, even though the presumed last step from protohypericin to hypericin was known to proceed spontaneously in vitro under illumination with visible light and in the presence of oxygen [69,70]. Since expression of the *Hyp-1* gene did not correlate with the sites of hypericin accumulation, and attempts to confirm the enzymatic activity of the Hyp-1 protein failed, doubts were raised with respect to the function of the *Hyp-1* gene product [71,72]. Ultimately, the conclusion that the Hyp-1 prtotein does not catalyze the proposed function were reached after a host of studies pertaining to *Hyp-1* mRNA accumulation under several conditions, as well as *in situ* and immunoblotting experiments were performed [73,74,75]. Differential expression studies, based on next generation sequencing of mRNA from dark gland-containing leaf margins and leaf blades devoid of dark glands in *H. perforatum* and related *Hypericum* species, identified three *PR-10-related* genes distinct from the *Hyp-1* gene [52]. These newly discovered sequences are specifically expressed in dark gland-containing tissues. Their coding sequences share only 46–50% amino acid identity with the Hyp-1 protein and 44–62% between each other. They were named phenol oxidative coupling proteins (*POCP*) 1 to 3, and assumed to perform the function formerly assigned to the Hyp-1 protein. To our knowledge, no experimental demonstration of their functions exists to date, but the crystal structures of Hyp-1 indicate the presence of a functional internal ligand binding site, similar to the one present in the sequence- and structure-related Bet v 1 birch allergen [71,76,77]. In addition, a PR-10-related protein performs the condensation of dopamine and 4-hydroxyphenylacetaldehyde in opium poppy, and suggests potential functions of *POCPs* in the oxidative coupling or cyclization of the octaketide intermediate in hypericin biosynthesis [78].

With respect to the initial formation of emodin anthrone, the suspected octaketide precursor of hypericin, the cloning of octaketide synthase (*OKS*), designated *HpPKS2*, was reported by Karppinen *et al.*, and its expression correlates with hypericin and pseudohypericin accumulation in tissues of *H. perforatum* [79]. *In situ* hybridization confirmed that *HpPKS2* expression is confined to dark glands, which are the sites of hypericin accumulation in *H. perforatum.* In addition, the encoded protein is able to produce octaketides from acetyl-CoA and seven malonyl-CoAs, but yields the derailed products *SEK4* and *SEK4b* and not the expected emodin anthrone [12,50,80,81]. It was speculated, that unidentified factors required for correct cyclization are missing from the in vitro reactions with *E. coli*-expressed and purified protein, similar to the situation for other *OKS* from *Aloe arborescens*, which were expected to yield chrysophanol anthrone, but produced only SEK4 and SEK4b [82,83]. So far, no polyketide cyclase which would catalyze the correct cyclization of the octaketide in the hypericin pathway has been identified, although the search for such a function in the pathway for the tetraketide olivetolic acid in *Cannabis sativa* had been successful [84].

Using the newly discovered development of dark glands in placental tissue of some *H. perforatum* lines, which is completely absent in other lines, Rizzo et al. used the overlap of differentially expressed genes (DEGs) between lines with and without dark glands, and DEGs between developmental stages with and without dark glands in the same line, to restrict the number of candidate genes involved in dark gland development and hypericin biosynthesis [14]. Together with metabolites identified by Ultra High Performance Liquid Chromatography-Electrospray ionization-High Resolution Mass Spectrometry (UHPLC-ESI-HRMS), which correlate with the presence of dark glands, a new biosynthetic pathway for hypericin was proposed. This new model suggests that penicilliopsin (10) is the first intermediate with two condensed octaketides instead of emodin dianthrone [14]. Since penicilliopsin links the two octaketide halves of hypericin via the same C-C bond as skyrin (13), this or a similar pathway seems to be supported by the metabolite profiling of 17 *Hypericum* species by Kimáková et al. [85]. In that study, skyrin and skyrin glucosides, but not emodin or emodin anthrone, are correlated with the presence of hypericin, suggesting skyrin as an intermediate in hypericin biosynthesis. A novel addition to the pathway proposed by Rizzo et al. is a gene encoding berberine-bridge enzyme (*BBE*), which was hypothesized to convert penicilliopsin to protohypericin, the last step of hypericin synthesis for which an enzyme is required [14]. Since this gene encodes an N-terminal signal peptide, the formation of the C-C double bond between the octaketide halves is likely to occur within the vacuole, in an ER-derived vesicle, or outside of the cell. In addition, Rizzo et al. confirmed the highly correlated expression of the *OKS* gene and the *POCP* genes identified by Sotak et al. with dark gland development, and identified novel candidate genes for the cleavage of the CoA-thioester bond, the cyclization of the octaketide, and hydroxylation of penicilliopsin to hydroxypenicilliopsin for the formation of pseudohypericin [12,13,14]. Furthermore, genes potentially involved in the transport of pathway intermediates across membranes (*ABC* transporter, *UDP-glucosyl transferase*, β-glucosidase, major facilitator protein for sugar import) and by vesicles (glutathione-S-transferases) were identified within the small set of DEGs highly upregulated during dark gland development [14]. A comparison of DEGs provided for *H. tomentosum* leaf tissues with and without dark glands and DEGs for dark gland development [14] show that almost all gene functions in the smaller set of DEGs occur in the larger set [13,14,52]. The only exceptions to this overlap are the early induced transcription factors *AGL6* and *MYB38*, which have been implicated in the differentiation of dark glands [14].

## 6. Hyperforin Biosynthesis

(+)-Hyperforin (27) (C_35_H_52_O_4_) is a polyprenylated acylphloroglucinol (PPAP) derivative with a phloroisobutyrophenone bicyclic core [86,87,88]. It is a mixture of interconverting and stably co-existing tautomers, yet it is unstable when exposed to light and oxygen [88,89,90,91]. This is due to the enolized β-dicarbonyl system present in phloroglucinols [91,92,93]. It is a fairly stable molecule in protic solvents and in in vivo systems [94]. Hyperforin occurs naturally in *H. perforatum* Linn. (Hypericaceae) in large amounts, (6.01 and 13.59 mg g^−1^ dry weight in leaves and flowers respectively), and in several other species, albeit at lower levels [95,96]. It is localized in pistils, flowers and fruits. In the development of the flowers, the hyperforin content increased from 2.47% to 8.48% (dry weight) from flower buds to fruits [97]. Such an increase was also reported by Maisenbacher and Kovar [98]. High concentrations of hyperforin accumulate in the translucent glands [48]. While extracts of *H. perforatum* have been used as an herbal supplement to treat depression as early as 1958, the determination that hyperforin was the metabolite responsible for the pharmaceutical affects was reported in 1971 [99,100]. The partial molecular structure was published four years later [86]. Further studies were able to focus on the stereochemistry of the molecule, and absolute configuration was ultimately resolved with the use of x-ray data crystallization techniques [87,101].

The evidence for the incorporation of intermediates from primary metabolism into hyperforin was ascertained via the use of feeding studies using labeled precursors. isobutyryl-CoA (17) has been determined to be one of the initial primary metabolite starter molecules in the biosynthesis of the hyperforin core structure. Furthermore, isobutyryl-CoA is derived from an α-ketoisovalerate intermediate (15) produced from a combination of pyruvate and valine (16) [88,102,103]. In many cases, the addition of precursors/substrates to cell/tissue cultures can enhance the production of specialized metabolites [104]. The addition of labelled valine, leucine and isoleucine to shoot cultures of *H. perforatum* revealed that valine was the most likely precursor [103]. Quantitative NMR spectroscopy analysis of *H. perforatum* cuttings immersed in (1-^13^C) glucose revealed that the dimethylallyl moieties were derived from the non-mevalonate pathway (MEP), and hence established the need for plastid-derived metabolites to be involved in supplying intermediates to the hyperforin biosynthetic pathway [88,105].

**Figure 7:**
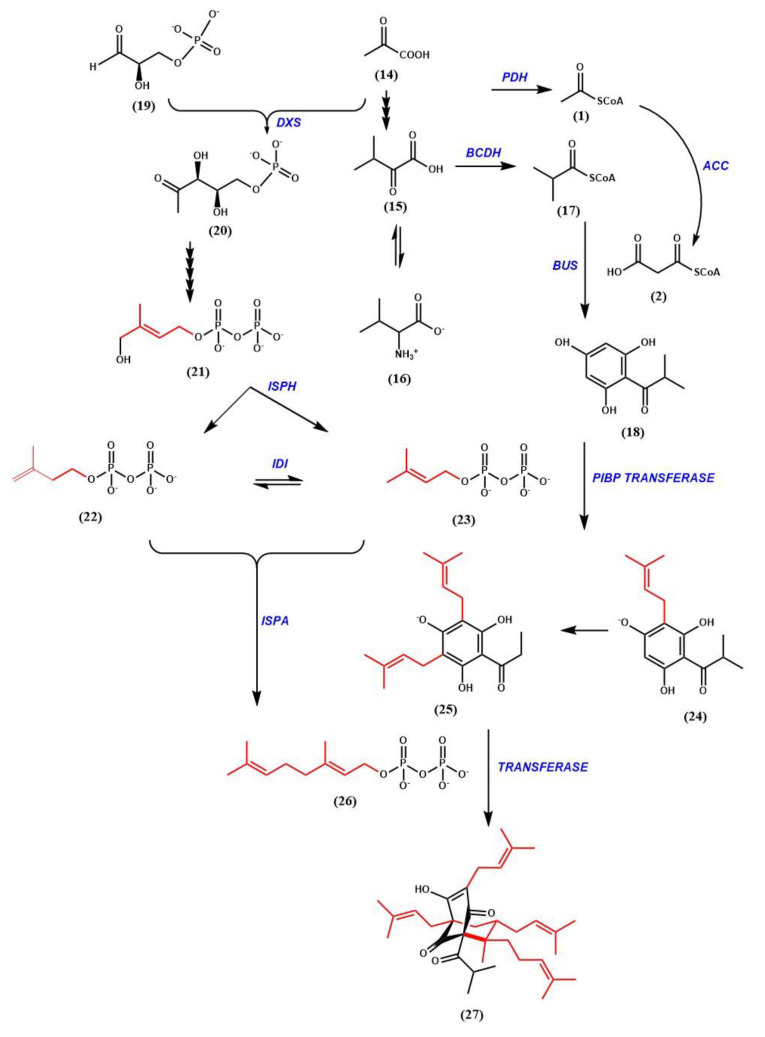
The hypothetical pathway for the biosynthesis of hyperforin: Prenylations are shown in red. Enzymes, represented in blue, are suggested by previous studies: *PDH*, pyruvate dehydrogenase; *ACC*, acetyl-CoA carboxylase; *BCDH*, branched chain α-keto acid dehydrogenase complex; *PIBP* transferase, phlorisobutyrophenone transferase; *BUS*, isobutyryl-CoA; *DXS*, 1-deoxy-D-xylulose-5-phosphate synthase; *ISPH*, 1-hydroxy-2-methyl-butenyl 4-diphosphate reductase; *IDI*, isopentenyl diphosphate isomerase; *ISPA*, Farnesyl pyrophosphate synthase.

The bicyclic structure of hyperforin suggests that it has elements with a meroterpenoid origin. The acyl phloroglucinol moiety is derived via a type III polyketide synthase (PKS)-type mechanism [88,106,107,108]. The hyperforin nucleus is formed by sequential condensation of one molecule of isobutyryl-CoA with three molecules of malonyl-CoA, catalyzed by Isobutyrophenone synthase (BUS) (Figure 7). It is known that type III PKS enzymes catalyze decarboxylative condensations of malonyl-CoA onto CoA-linked starter molecules, which are often followed by cyclization within the enzyme active site to generate scaffolds [109,110]. Similar observations were made in *Humulus lupulus* by Okada et al. and Clark et al. [111,112]. Klingauf et al. observed the presence of three type III PKS enzymes in the cell cultures of *Hypericum calycinum* [107]. These three enzymes each preferred a different substrate, and did not produce identical products. When the cell-free extracts from the cell cultures were incubated with isobutyryl-CoA and malonyl-CoA, phlorisobutyrophenone (18) was formed. The enzyme catalyzing this reaction was identified as BUS. A functionally similar enzyme in glandular hairs of hop cones participates in the biosynthesis of bitter acids [106]. Two acylphloroglucinol cores, namely phlorisovalerophenone (PIVP) and phloroisobutyrophenone (PIBP), are formed by Claisen condensation, but differ in substrate and enzyme specificities. While PIVP uses isovaleryl-CoA in the presence of the enzyme valerophenone synthase (VPS), PIBP uses isobutyryl-CoA in the presence of BUS, resulting in the production of adhyperforin and hyperforin, respectively [106].

The PKS product (hyperforin core), undergoes stepwise prenylation to ultimately produce hyperforin. No enzyme activity has yet been identified that utilizes phloroglucinol as a prenyl acceptor. The preferred prenyl acceptor was phlorisobutyrophenone (18). A distinct prenyltransferase activity has been identified as participating in the first prenylation step of the hyperforin core. Product formation can also be achieved in the presence of IPP (22), which was attributed to IPP isomerase activity. However, no enzyme activity could be determined in which GPP (26) and FPP were used as prenyl donors [113]. This would suggest that the prenylation steps requiring these substrates are catalyzed by a different enzyme. Prenyl transferases with similar properties were also reported in *Humulus lupulus* and in *Cannabis sativa*, in the biosynthesis of bitter acids and cannabinoids, respectively [112,114,115]. The prenylation of phlorisobutyrophenone (18) followed Michaelis-Menten kinetics. The *K*_M_ value for the acceptor was 0.52 mM, and 0.46 mM for DMAPP [113]. The transferase activity was dependent on a divalent cation with Fe^2+^ (*K*_M_ = 3.8 mM) as the most efficient cofactor, and showed a broad pH optima from 6.5 to 8.5. This is the first soluble prenyltransferase which prefers Fe^2+^ as a divalent cation over the more commonly used Mg^2+^ or Mn^2+^ ions. Another round of prenylation occurs, involving geranyldiphosphate as a prenyl donor. Consequently, triple electrophilic substitution of the unsubstituted hyperforin nucleus involves two DMAPP (23) units and one GPP molecule [88]. The ring closure of the bicyclic system is triggered by electrophilic attack of a third DMAPP on the 2’/3’ double bond of the geranyl chain, resulting in the production of hyperforin. The stage at which the ring closure occurs is open to debate [116].

## 7. Perspectives of Engineering the Biosynthetic Pathways of Relevant Compounds from *H. perforatum* in Microorganisms and Plants

The active compounds produced by *H. perforatum*, especially hypericin and hyperforin, are of great importance to the pharma industry, and the elucidation of their biosynthetic pathways would lay the foundations for engineering the production of these compounds in microorganisms. Nevertheless, the engineering of such compounds is a complex problem and multiple aspects must be taken into account. Some of the compounds discussed here are cytotoxic, and they are normally compartmentalized inside specialized organs (glands), like in the case of hypericin. This implies that the sole knowledge of the genes involved in the biosynthesis of these molecules is not enough for their large-scale production. Engineered microorganisms could die soon after starting the biosynthesis of molecules such as the naphtodianthrones, because they cannot store them inside dedicated glands. There is at least one case reported in the literature of a fungus producing hypericin, and several other studies reporting the enhancement of hypericin production in the presence of arbuscular mycorrhizae or in response to the infection with fungal pathogens [117,118,119,120]. Nevertheless, these organisms are endophytic fungi that rely on the compartmentalization carried out by the plant. For this reason, a realistic perspective for the large scale production of bioactive compounds in SJW should rely on the biosynthesis genes, as well as on the genes involved in the differentiation of dark glands (*AGL6*, *MYB38*), and the translocation and secretion mechanisms (GST). In this way, the generation of new varieties with hyperactivated (or knocked out) glands could be possible, and the production of these highly valuable compounds could be carried out *in planta*.

Possible means of synthesizing hyperforin in microorganisms have been attempted [121,122]. However, a total biosynthesis of hyperforin using a bacterial system has not yet been successful. On the other hand, the production of phlorisovalerophenone, a key intermediate of humulone and adhyperforin biosynthesis, has been achieved in *E. coli* [102]. In this system, isovaleryl-CoA was produced employing a pathway involving hydroxyl-3-methylglutaryl CoA (HMG-CoA), an enzyme that is known to be involved in the mevalonate pathway leading to the biosynthesis of isoprenoids [123]. The enzymes acetyl-CoA acetyltransferase and HMG-CoA synthase were used to yield HMG-CoA. Further steps included the addition of HMG-CoA dehydratase, MG-CoA decarboxylase and DMA-CoA reductase to produce isovaleryl-CoA. After generating isovaleryl CoA, the type III PKS valerophenone synthase that was introduced into an engineered *E. coli* strain. The system ultimately produced phlorisovalerophenone at a concentration of 6.4 mgL^-1^. This system utilizes glucose as the main carbon source for entry into the pathway leading to PIVP, which suggests that an economical biosynthetic production of these compounds is possible.

## 8. Conclusions

In recent years, *Hypericum* research has experienced a resurgence of scientific interest due to the newly discovered potential of its bioactives, and their abilities to be utilized as an anticancer medicine and a possible treatment for neurodegenerative diseases. Hypericin and hyperforin are the metabolites with the highest value for the pharmaceutical industry. Therefore, the engineering of their biosynthesis in orthologous systems (especially microorganisms) would be advantageous for the development of new drugs. Challenges related to compound toxicity and storage may slow the current pace of advancement, however this may well be overcome by utilizing different strains of microorganisms or even by using transformed plant systems.

The future of *H. perforatum* research as well as other related species will heavily depend on the use of large-scale omics methodologies for the identification of putative genes involved in the regulation and biosynthesis of pharmaceutically important compounds. Already, the recent discoveries using dark gland-bearing tissues have provided many sequences to test. While a few studies have been published regarding stable transformation of *Hypericum*, they are inconsistent and not yet optimized [124,125]. Ongoing research using overexpression and knockout technologies, such as CRISPR-Cas9, will allow researchers to test the current biosynthesis models in a systematic fashion. Functional validation will open the doors to many promising possibilities and novel lines of research for the *Hypericum* community. The discovery of putative transcription factors correlated with dark gland development may be used in transgenic overexpression and knockout lines to further tease apart the links between gland structure and metabolism. Overall, the future of *H. perforatum* is full of new possibilities that, with the help of modern synthetic and molecular biology techniques, will lead to more diversified and powerful products based on SJW.
